# Elective Neck Dissection Strategies Guided by AJCC-8 Depth-of-Invasion (DOI) in cT1–T2N0 Oral Cavity Cancer—A Systematic Review

**DOI:** 10.3390/cancers18040697

**Published:** 2026-02-20

**Authors:** Nishath Sayed Abdul, Sahana Shivakumar, Lulwah Alreshaid, Ankur Jethlia, Honey Lunkad, Maria Maddalena Marrapodi, Gabriele Cervino, Giuseppe Minervini

**Affiliations:** 1 Faculty of Oral Pathology, Department of OMFS and Diagnostic Sciences, College of Medicine and Dentistry, Riyadh Elm University, Riyadh 11681, Saudi Arabia; nishathsayed@riyadh.edu.sa; 2 Department of Public Health Dentistry, People’s College of Dental Sciences and Research Centre, People’s University, Bhopal 462037, Madhya Pradesh, India; sahana20579@gmail.com; 3 Department of Restorative and Prosthetic Dental Sciences, College of Dentistry, King Saud bin Abdulaziz University for Health Sciences (KSAU-HS), Riyadh 11426, Saudi Arabia; reshaidl@ksau-hs.edu.sa; 4 King Abdullah International Medical Research Center, Ministry of National Guard—Health Affairs, Riyadh 11481, Saudi Arabia; 5 Dental Services, King Abdulaziz Medical City, Ministry of the National Guard—Health Affairs, Riyadh 11426, Saudi Arabia; 6 Department of Maxillofacial Surgery and Diagnostic Sciences, Diagnostic Division, College of Dentistry, Jazan University, Jazan 45142, Saudi Arabia; jethliaankur@yahoo.co.in; 7 Department of Prosthetic Dental Sciences, College of Dentistry, Jazan University, Jazan 45142, Saudi Arabia; drhoney_82@yahoo.co.in; 8 Department of Woman, Child and General and Specialized Surgery, Pediatric Unit, University of Campania “Luigi Vanvitelli”, 80138 Naples, Italy; 9 Department of Biomedical and Dental Sciences, Morphological and Functional Images, University of Messina, G. Martino Polyclinic, 98125 Messina, Italy; gcervino@unime.it; 10 Multidisciplinary Department of Medical-Surgical and Odontostomatological Specialties, University of Campania “Luigi Vanvitelli”, 80138 Naples, Italy; giuseppe.minervini@unicampania.it

**Keywords:** oral cavity cancer, depth of invasion, elective neck dissection, AJCC-8, occult metastasis, survival

## Abstract

Background/Objectives: Clinically node-negative (cN0) neck management in cT1–T2 oral cavity squamous cell carcinoma continues to be a subject of controversy. The eighth edition AJCC has incorporated depth of invasion (DOI) as a significant factor in staging and consideration for possible neck dissection. Establishment of accurate DOI thresholds and their clinical relevance is crucial to maximize oncological outcomes with reduced unnecessary morbidity. Methods: A comprehensive analysis of clinical research assessing elective neck dissection (END) techniques in oral cavity cancers classified by DOI in cT1–T2N0 patients was carried out. The included studies reported occult nodal metastasis rates, overall survival, disease-specific survival, disease-free survival, and regional control. Results: With hazard ratios favoring END for overall survival (HR 0.64; 95% CI 0.45–0.92) and disease-free survival (HR 0.45; 95% CI 0.34–0.59), elective neck dissection provided advantages in both survival and regional control. In a national registry, DOI ≥ 5 mm independently raised the risk of nodal failure (HR 2.099; 95% CI 1.346–3.271), while END enhanced neck control in comparison to observation (HR 1.749; 95% CI 1.141–2.680). With ROC-derived cut-offs like 4.59 mm producing positive predictive values for nodal metastasis up to 41.7%, diagnostic thresholds clustered around 4 mm. Conclusions: Under DOI guidance, elective neck dissection consistently showed oncologic benefit, with practical thresholds convergent around 4 mm for sites in the mixed oral cavity and 3 mm for high-risk subsites. The synthesized results confirmed that DOI is the primary determinant of END when combined with histopathologic and subsite-specific risk factors.

## Simple Summary

There is often debate regarding whether to retain or remove lymph nodes in cases of early-stage oral cancer when they are not swollen. The 8th edition of the American Joint Committee on Cancer (AJCC) identifies the “depth of invasion” of a tumor as an indicator of potential metastasis or hidden spread; however, the optimal threshold for intervention remains uncertain. We have systematically reviewed relevant clinical studies that use “depth of invasion” to guide neck dissection in the early stages of oral cancer. The variables extracted from the data included hidden node rates and survival outcomes. Our qualitative synthesis indicates that the removal of neck nodes with a depth of 3 to 4 mm correlates with improved rates of disease control and survival. Avoiding needless surgical procedures is crucial when dealing with superficial tumors. These results provide plausibility to a selective, depth-guided approach to neck treatment, which can help guideline committees and doctors customize surgical plans for specific patients.

## 1. Introduction

Oral cavity squamous cell carcinoma (OCSCC) remains a substantial contributor to the morbidity and mortality rates associated with head and neck cancers on a global scale. Survival outcomes are significantly impacted by the existence of cervical lymph node metastasis at the point of diagnosis or during later follow-up evaluations [1]. The management of the clinically node-negative (cN0) neck in cases of early primary tumors (cT1–T2) has persistently been a subject of contention. This is attributed to the inconsistencies in the probability of occult metastasis among various subsites and tumor biology, in addition to the functional complications tied to elective neck dissection that necessitate justification through a corresponding oncologic advantage [2].

The eighth edition of the American Joint Committee on Cancer (AJCC-8) integrated DOI into the T-classification for oral cavity primaries, thereby establishing an anatomical marker for infiltrative behavior and nodal risk that had been recognized in multiple institutional investigations [3]. By linking T-upstaging to DOI categories, AJCC-8 provided a standardized framework for risk stratification. However, it abstained from defining explicit treatment thresholds for END, necessitating that clinicians harmonize population-level staging with decisions pertaining to individual patients [3].

The DOI functioned mechanistically as an intermediate between the tumor–host interface and lymphovascular access, and empirically as a linear predictor of occult nodal metastasis and regional failure. The risks associated with DOI were seen to accrete asymmetrically over the different oral subsites, such as the floor of the mouth, oral tongue, gingiva, and buccal mucosa [4]. Further evidence revealed that specific histopathologic features—namely, perineural invasion (PNI), lymphovascular invasion (LVI), grade, and worst pattern of invasion—modulated nodal risk at a given depth, thus justifying composite risk models in which DOI was an important but not absolute feature [5].

Preoperative determination of DOI by intraoral ultrasound, MRI, or CT improved the potential for anticipatory decision-making; however, each modality revealed constituent bias and calibration requirements with respect to the pathological DOI. Ultrasound was used mainly for real-time, chairside evaluation in the oral tongue, whereas MRI was used more commonly in situations where submucosal extension or base of the tongue invasion was a concern [5].

The clinical dilemma pertained to the identification of the thresholds at which elective neck dissection yielded a net advantage relative to observation or alternative staging methodologies, including sentinel lymph node biopsy (SLNB). This identification required an assessment of subsite heterogeneity, comorbid conditions, rehabilitation needs, and systemic resources [6]. Literature evidence shows consistent oncologic benefits of timely at-risk neck management, but the exact threshold at which benefit outweighed morbidity was determined by the distribution of DOI, the risks inherent to local therapies, and the presence of effective salvage strategies within surveillance protocols [7].

Decision-analytic modeling identified actionable thresholds based on an estimated probability of occult metastasis of approximately 20%, translating epidemiological gradients of depth of invasion into practical guidelines. It included universal triggers near 4 mm and subsite-specific thresholds around 3 mm for high-risk subsites. However, external validation across diverse settings, imaging modalities, and patterns of adjuvant therapy demonstrated substantial variability [8,9]. In this context, the present review sought to examine clinical human studies of cT1–T2N0 OCSCC.

## 2. Materials and Methods

### 2.1. Eligibility Criteria and Review Design

The PECOS framework used in this systematic review was developed in accordance with the PRISMA 2020 reporting guidelines [10] to provide methodological transparency, reproducibility, and scientific integrity. The study population included patients with cT1–T2 clinically node-negative OCSCC, regardless of age, sex, or geographic location. The exposure of interest was the determination of DOI according to AJCC-8 criteria based on either histopathologic assessment or validated preoperative imaging modalities. Comparators consisted of neck management strategies, namely elective neck dissection. Outcomes of interest included overall survival, disease-specific survival, disease-free survival, regional control, and incidence of occult nodal metastasis, with additional analyses considering treatment-effect heterogeneity, morbidity, and decision-analytic endpoints when appropriate. Eligible study design included prospective, retrospective, nonrandomized studies, and RCTs conducted in human subjects.

### 2.2. Inclusion and Exclusion Criteria

The review included primary clinical studies that compared patients with cT1–T2N0 OCSCC, for whom elective neck dissection approaches were directed or stratified according to AJCC-8 DOI cutoffs. Included were prospective cohorts, retrospective cohorts, and RCTs reporting quantitative data. All oral cavity subsites (tongue, floor of the mouth, buccal mucosa, gingiva, hard palate, alveolar ridge, retromolar trigone) were eligible for consideration. Included studies were required to report rates of survival, neck control, or occult metastasis by DOI. Exclusion criteria included systematic reviews, meta-analyses, editorials, letters, protocols, animal studies, and in vitro studies. Studies with oropharyngeal, hypopharyngeal, or laryngeal primaries were excluded unless oral cavity results were separately reported. Omitted were articles lacking original clinical data, those lacking DOI-stratified neck management, and those lacking outcomes of interest.

### 2.3. Database Search Protocol

The search strategy was formulated across six databases—PubMed, Embase, Scopus, Web of Science, Cochrane Library, and Google Scholar—utilizing Boolean operators, free-text keywords, and controlled vocabulary, including MeSH and Emtree terms. There were no restrictions regarding publication date, language, or age demographics (Table 1).

### 2.4. Data Extraction Protocol

Data extraction was conducted in duplicate by two reviewers independently with a pre- standardized template in place to ensure methodological uniformity. The items extracted were: identifiers for studies (author, year, country, setting), study design, sample size, patient demographics, distribution of tumor subsites, basis of staging (clinical vs. pathological T), methods of DOI assessment (histopathology, ultrasound, MRI, CT), DOI thresholds and strata, methods of neck management (elective neck dissection, sentinel lymph node biopsy, observation), reference standards for nodal status, and primary outcomes (occult nodal metastasis rate, overall survival, disease-specific survival, disease-free survival, regional control). Additional extracted variables were statistical models used (Cox regression, logistic regression), methods of cut-point determination (ROC, Youden index, pre-specified), treatment–effect heterogeneity by subsites or histopathological factors (grade, PNI, LVI), morbidity related to neck management, and decision-analytic endpoints (number needed to treat, net benefit curves). All differences were resolved by consensus, and any missing data were addressed by full-text review.

### 2.5. Bias Assessment Protocol

Risk of bias was established using the ROBINS-I V2 tool [11] for nonrandomized studies and the Cochrane RoB 2.0 tool [12] in RCTs.

### 2.6. GRADE Assessment

The confidence in the evidence from the included studies was assessed according to the GRADE approach [13], in combination with the results presented by the ROBINS-I tool [11] for nonrandomized trials and the Cochrane RoB 2.0 tool [12] for RCTs.

## 3. Results

### 3.1. Studies Included

**Figure 1:**
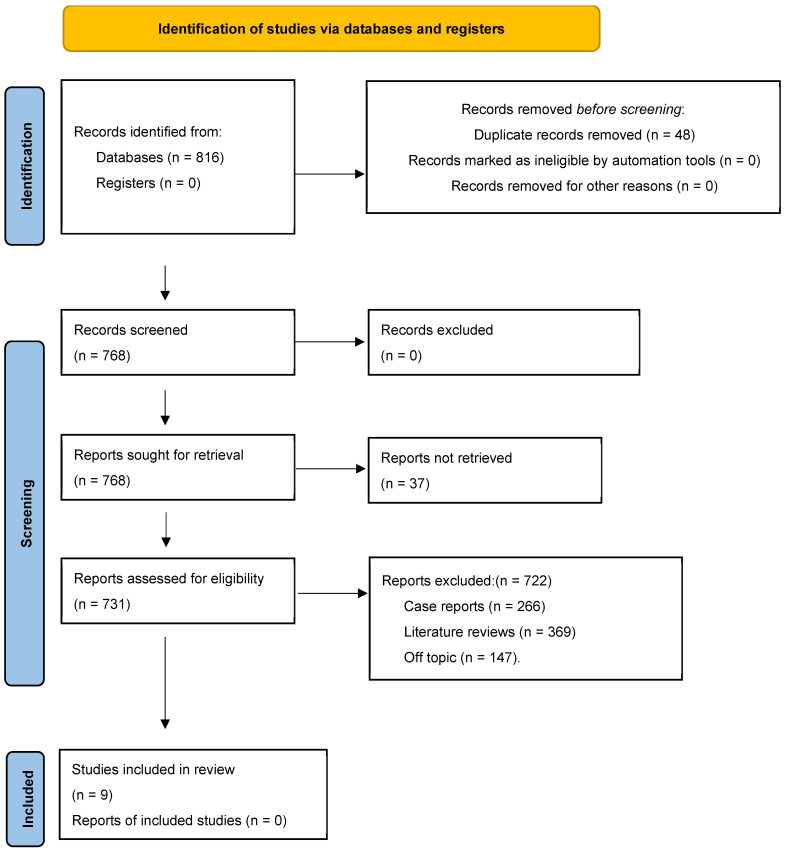
Study selection process for the review.

A total of 816 records were identified from all the databases, of which 768 records were screened following the removal of 48 duplicates. Of these, 37 reports were not retrieved, leaving 731 full-text articles to be assessed for eligibility. Among them, 722 articles were excluded (266 case reports, 309 literature reviews, and 147 not relevant to the review question) (Figure 1). The final inclusion for synthesis included 9 studies [5,7,14,15,16,17,18,19,20]. The protocol has been registered in the International Prospective Register of Systematic Reviews database (PROSPERO: CRD420261301180). 

### 3.2. Bias Levels Observed

**Figure 2:**
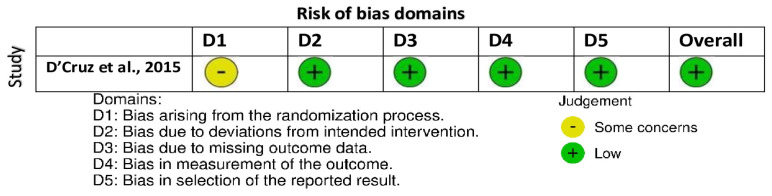
Bias assessment using RoB 2.0 tool [16].

**Figure 3:**
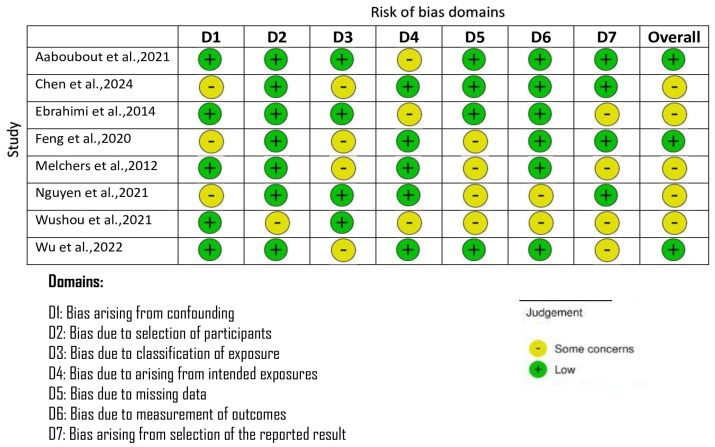
Bias assessment using ROBINS-I tool [5,7,14,15,17,18,19,20].

The only RCT included had a low overall risk of bias but reported some issues under the randomization category [16] (Figure 2). However, the various nonrandomized studies evaluated with the ROBINS-I tool had low bias across confounding, selection, and outcome measurement domains [14,20] (Figure 3). However, there were moderate issues with such aspects as the categorization of interventions and missing data management in registry-based or retrospective cohorts [5,7,15,17,18,19].

### 3.3. Demographic Variables Assessed

The location of the studies included single-center cohorts from the Netherlands [14], India [16], New Zealand [7], and China [20]; national registries from Taiwan [15] and the United States [19]; a multi-institution, multi-country observational cohort [17]; and a transnational two-center series from the United States and China [5] (Table 2). Enrollment sizes varied from small institutional cohorts (*n* = 70 [7]) and small single-center series (n = 178 [20]; n = 212 [18]; n = 222 [14]) to large national or multinational datasets (n = 4723 [15]; n = 5752 [19]; n = 3149 [17]). Age profiles were typical of early–older adult disease, with mean or median ages clustering in the 5th–7th decades (mean 48 y [16]; median 53 y [17]; median ~61.5 y [18]; mean ~62 y [5]; mean 62.0 y [19]; mean 65 y [7]). Sex distributions were male-predominant where reported (e.g., 4205:518 [15]; 2074:1075 [17]; 138:84 [14]; 119:93 [18]; 40:30 [7]). Follow-up windows gave time-to-event analyses across studies, including medians around 39 months in the randomized comparison [16], ~40 months in the staging cohort [17], ~55 months in a single-center series [7], ~56.5 months in an observation subgroup [21], ~62.4 months mean in a population registry [19], and protocolized assessment to ≥24 months in a bi-institutional cohort [5].

### 3.4. Technical Specifications Assessed

Anatomical scope included major oral cavity subsites (gingiva, tongue, floor of mouth, retromolar, lip, buccal) in the majority of datasets [5,7,14,15,17,18,19], and one dataset was limited to the oral tongue to permit ultrasound-based DOI stratification [20] (Table 3). Staging bases varied: several cohorts were pathologic pT1–T2 under AJCC-8 definitions [14,17,18], others were clinical cT-based (cT1–T2 in a randomized trial [16], cT2N0 in a national registry [15], cT1N0 across institutional series [7,18,20], and cT1N0 in a population registry [19]). DOI ascertainment typically relied on pathology using the AJCC-concordant reconstructed mucosal plane [14,17,18], with preoperative intraoral ultrasound enabling real-time DOI triage in the oral tongue [20]; one population registry lacked DOI capture [19]. DOI distributions were reported as medians/means or bins: median ~4.5 mm with binning at ≤4 mm vs. >4 mm [14], median 6.0 mm with ROC-optimized separation [18], median by pT of roughly 5/9/13.5/15 mm across pT1–pT4 [17], subsite-specific bins at <2, 2–3, 3–4, 4–5, ≥5 mm [5], and ultrasound DOI <4 mm vs. ≥4 mm for triage [20].

Decision thresholds that explicitly guided END included >4 mm anchored to an occult metastasis risk exceeding ~20% [14], ≥5 mm derived from cut-point optimization in cT2N0 disease [15], AJCC-8 staging bands at 5 mm and 10 mm used for prognostic discrimination [17], subsite-specific triggers around ≥3 mm for high-risk sites and ≥4 mm for others [5], an ROC/Youden-driven value of 4.59 mm pragmatically rounded to 4 mm [18], and a conservative policy threshold at ≥3 mm in a stage I cohort [7]; two datasets did not allocate treatment by DOI (trial allocation [16] and a registry without DOI [19]). Models frequently integrated histopathologic and clinical covariates (grade [5,7,15,20,21], margins [15], comorbidity [15], LVI [7,18], PNI [7,18,20], and diameter [14]).

Neck management arms contrasted END (often selective, ipsilateral or bilateral) with observation or delayed therapeutic dissection [7,18,20], with adjuvant therapy per practice patterns in a national dataset [15]. Reference standards for nodal status included pathologic yields in END necks (median ~27 nodes [15]), ≥2-year follow-up to verify cN0 in observed patients [18], and registry survival outcomes where pathologic detail was variably available [19]. Primary endpoints spanned overall and disease-specific survival [16,17,19], neck control [15,20], occult nodal metastasis and regional recurrence [7,14,18], and subsite-specific 2-year nodal risk tables to inform thresholding [5]

Large heterogeneity in the ascertainment and thresholding of DOI across included studies was noted. DOI was often based on postoperative pathology, using reconstructed mucosal plane protocols in many series, although several relied on registry-extracted pathology, at least one study used intraoral ultrasound, and other studies were not guided by DOI or did not report DOI at all. Therefore, no cross-study standardization or recalibration of DOI was attempted, since primary reports provided neither paired measurements nor calibration factors needed to perform a valid adjustment. DOI was synthesized as reported, with an explicit statement of the modality used. Thresholds guiding elective neck dissection ranged from ≥3 mm through to approximately 4–4.6 mm to ≥5 mm, with several studies employing subsite-specific cut-points. This heterogeneity and variability limit the generalizability of any single DOI threshold and restrict generalizability across institutions. As such, the findings should be viewed as supporting DOI as a risk-stratification variable with context-dependent action thresholds sensitive to modality and subsite rather than advocating a single globally transferable value of DOI.

### 3.5. Outcomes and Quantitative Observations

Across treatment-effect evaluations, elective treatment of the cN0 neck conferred clinically meaningful advantages where tested, with hazard ratios favoring END for overall survival (HR 0.64; 95% CI 0.45–0.92) and disease-free survival (HR 0.45; 95% CI 0.34–0.59) in a randomized comparison of early oral cavity cancer managed without DOI guidance [16] (Table 4). In a cT2N0 national registry anchored to pathologic DOI, observation versus END was associated with inferior neck control (HR 1.749; 95% CI 1.141–2.680), while DOI ≥ 5 mm independently increased failure risk (HR 2.099; 95% CI 1.346–3.271) after adjustment for grade, margins, adjuvant therapy, and comorbidity [15].

At the diagnostic-threshold level, occult metastasis risk crossed the traditional 20% actionability boundary around ~4.3 mm, supporting a pragmatic END trigger at >4 mm [14], and an ROC/Youden procedure yielded a 4.59 mm cut-off with a positive predictive value for nodal positivity near ~41.7% in an observed subgroup, justifying a rounded 4 mm rule in clinical use [18]. Preoperative ultrasound stratification provided actionable prognostic separation: for ultrasound DOI ≥ 4 mm, regional failure was ~20.8% under observation versus ~6.2% with END (log-rank *p* ≈ 0.031) and disease-specific survival improved from ~67% to ~86% at 5 years (*p* ≈ 0.033), while <4 mm strata showed no material decrement under observation, enabling selective avoidance of END [20]. Population-level analyses further supported survival gains with END in cT1N0 disease (OS HR 0.708; *p* < 0.001; DSS *p* = 0.0004) despite the absence of DOI capture, consistent with a general benefit to treating at-risk necks proactively [19].

## 4. Discussion

The management of the cN0 neck in the context of early OCSCC has increasingly highlighted the significance of incorporating anatomical, pathological, and imaging-based risk factors to enhance elective treatment protocols. The specific site-related variations in the probability of nodal metastasis have been persistently noted, with certain subsites, particularly the floor of the mouth and the base of the tongue, demonstrating an increased likelihood of occult spread at shallower depths when contrasted with buccal mucosa or gingiva, thereby supporting the notion of intervention thresholds tailored to particular subsites [21]. Alongside anatomical considerations, SLNB has emerged as a feasible alternative to END, presenting the benefit of diminished morbidity while ensuring oncological safety in appropriately chosen cases; however, challenges persist concerning standardization, accessibility, and incorporation into standard practice [22].

This review showed that DOI is an effective predictor of nodal metastasis and regional failure, with thresholds of 3–4 mm determining the boundary for elective neck treatment. The results reminded us that DOI-guided strategies should supplant homogeneous elective strategies, especially when combined with histopathologic modifiers like grade, perineural invasion, and lymphovascular invasion. Future clinical practice guidelines might take advantage of the incorporation of subsite-specific DOI triggers, with more liberal cut-offs for subsites at increased risk like the floor of the mouth. The role of preoperative ultrasound for real-time DOI stratification suggested the potential of minimally invasive triage devices for optimizing surgical decision-making. Continued investigation should aim to validate DOI-based algorithms in prospective, multi-institutional trials, including decision-analytic modeling, and assessment of patient-centered outcomes like morbidity, quality of life, and cost-effectiveness.

The importance of DOI as an essential threshold variable has been validated, with several clinical studies reporting evidence for a cutoff of 4 mm for occult metastasis probability prediction in situations where elective neck dissection is clinically indicated [23]. However, DOI must be regarded in conjunction with other variables; i.e., histopathologic variables like perineural invasion, lymphovascular invasion, and tumor grade individually contribute to recurrence risk stratification and correlate with DOI in models of prognosis [24]. Furthermore, measurement of DOI has become increasingly complex, with imaging modalities like MRI and ultrasound demonstrating improved reliability for preoperative assessment; however, there are discrepancies between such modalities and the gold standard of histopathologic examination, and accurate diagnosis is thus necessary in clinical practice [25].

Uncommon tumors like primary oral mucosal melanoma highlight the heterogeneity of oral cancers, where DOI cannot always be represented by common squamous risk models, and diagnosis-specific models are needed [26]. In common OCSCC, subsite-by-subsite analysis, e.g., buccal mucosa carcinomas, shows multilayered DOI correlations with nodal metastasis, again highlighting the need for accurate models rather than uniform cut-offs [27]. Although patient selection is still essential to guarantee safety and effectiveness, SLNB validation studies also support its use in early oral malignancies [28]. Independent of DOI, adverse histopathological characteristics like tumor budding and the worst invasion pattern have been linked to worse outcomes, indicating that composite models could more accurately represent biologic aggressiveness [29]. Likewise, resection margins and metastatic patterns outweigh DOI in terms of prognostic importance for oral mucosal melanomas, which show unique survival determinants [30].

Preoperative DOI assessment is critical for END, as neck treatment is typically determined prior to surgical intervention. Intraoral ultrasound and MRI DOI measurements can help to stratify risk; however, they are susceptible to systematic measurement error and may not be directly interchangeable with histopathologic DOI due to factors such as tissue shrinkage, plane reconstruction, and operator dependence. Preoperative DOI criteria should be viewed with caution and modified based on institutional pathological correlations, rather than being treated as definitive thresholds. Ultrasound-based DOI shows promise for real-time triage in oral tongue cancers. A DOI of 4 mm or more assessed preoperatively may mean END, while lower values may mean that low-risk subsites need to be watched. Preoperative DOI, subsite, imaging modality, and clinical risk factors offer an optimal basis for personalized decision-making.

The review is strengthened by recent cohort investigations that have continued to assess the need for END in small tumors, especially in tongue squamous cell carcinoma [31]. Although actual implementation differs by practice setting, national registry studies have operationalized elective neck treatment thresholds by codifying DOI into AJCC-8 staging bands at the population level [32]. Additionally, surgical margin status and elective neck treatment continue to be major concerns in maxillary OSCC, in which loco-regional disease control dictates long-term survival outcomes [33]. Predictive nomograms including DOI, histopathologic risk factors, and patient covariates have been constructed to predict nodal recurrence-free survival and are an early step towards precision oncology [34]. Specialist group position statements have further reiterated the recommendation for individualized, evidence-based cN0 neck treatment, in favor of treatment based on a risk-adapted strategy according to DOI, subsite, and pathologic characteristics rather than a policy of treatment for all [35].

This is a systematic review with narrative synthesis. No meta-analysis was conducted as measures to report DOI varied. Also, outcomes reported used different designs and effect measures. Pooled results obtained from such heterogeneous data could result in misleading averages.

We did not perform a meta-analysis because the included studies were too different from each other to combine safely. They used different ways to measure and report depth of invasion (pathology vs. ultrasound vs. registry data), applied different DOI cut-offs (3 mm to 5 mm and subsite-specific thresholds), and reported outcomes using different designs and effect measures. Pooling such heterogeneous data could give a misleading “average” effect, so we used a narrative synthesis to present the evidence more accurately.

### Limitations

The observed evidence in this review was constrained by the predominance of retrospective data, heterogeneous methods to calculate DOI, inconsistencies in thresholds across institutions, and sparse documentation of morbidity outcomes. Similarly, registry data sets were not defined at the pathological level, and only a few studies employed formal decision-analytic models.

## 5. Conclusions

Elective neck dissection techniques employing DOI in cT1–T2N0 oral cavity cancer offered a rational, evidence-based model that weighed oncologic gain against treatment morbidity. DOI cut points clustered at approximately 4 mm for universal use, with subsite-specific modifications and histopathologic risk modifiers adding specificity to clinical judgment. The results validated selective, DOI-directed elective neck treatment as a mainstay of modern management, while reaffirming the necessity of ongoing prospective validation to maximize patient benefit.

## Figures and Tables

**Table 1:** Search strings across databases.

Database	Search String
PubMed	(“oral squamous cell carcinoma”[tiab] OR “oral cavity cancer”[tiab] OR “tongue cancer”[tiab] OR “floor of mouth”[tiab] OR “buccal mucosa”[tiab] OR “gingiva”[tiab] OR “retromolar trigone”[tiab]) AND (“depth of invasion”[tiab] OR “tumor thickness”[tiab] OR “invasion depth”[tiab]) AND (“neck dissection”[tiab] OR “elective neck dissection”[tiab] OR “selective neck dissection”[tiab] OR END[tiab]) AND (AJCC[tiab] OR “8th edition”[tiab] OR TNM[tiab]) NOT (review[pt] OR editorial[pt] OR case reports[pt] OR animals[mh] NOT humans[mh])
Embase	(‘oral squamous cell carcinoma’/exp OR ‘oral cavity cancer’:ab,ti OR ‘tongue cancer’:ab,ti OR ‘floor of mouth’:ab,ti) AND (‘depth of invasion’:ab,ti OR ‘tumor thickness’:ab,ti OR ‘tumor thickness’:ab,ti) AND (‘elective neck dissection’:ab,ti OR ‘selective neck dissection’:ab,ti OR END:ab,ti) AND (‘ajcc 8th edition’:ab,ti OR TNM:ab,ti) NOT ([animal]/lim NOT [human]/lim) NOT ([review]/lim)
Scopus	TITLE-ABS-KEY((“oral squamous cell carcinoma” OR “oral cavity cancer” OR “tongue cancer” OR “floor of mouth” OR “gingiva” OR “buccal mucosa” OR “retromolar trigone”) AND (“depth of invasion” OR “tumor thickness” OR “invasion depth”) AND (“elective neck dissection” OR “selective neck dissection” OR END) AND (“AJCC 8th” OR TNM)) AND NOT TITLE-ABS-KEY(review OR “in vitro” OR animal)
Web of Science	TS = (“oral squamous cell carcinoma” OR “oral cavity cancer” OR “tongue cancer” OR “gingiva” OR “floor of mouth” OR “buccal mucosa”) AND TS = (“depth of invasion” OR “tumor thickness”) AND TS = (“elective neck dissection” OR “selective neck dissection” OR END) AND TS = (“AJCC 8th edition” OR TNM) NOT TS = (review OR “in vitro” OR animal)
Cochrane Library	(“oral squamous cell carcinoma” OR “oral cavity cancer” OR “tongue cancer” OR “floor of mouth” OR “gingiva” OR “buccal mucosa”) AND (“depth of invasion” OR “tumor thickness”) AND (“elective neck dissection” OR “selective neck dissection” OR END) AND (“AJCC” OR “TNM” OR “8th edition”)
Google Scholar	(“oral squamous cell carcinoma” OR “oral cavity cancer” OR “tongue cancer” OR “floor of mouth” OR “buccal mucosa” OR gingiva OR “retromolar trigone”) AND (“depth of invasion” OR “tumor thickness”) AND (“elective neck dissection” OR “selective neck dissection” OR END) AND (AJCC OR TNM OR “8th edition”) —review —“systematic review” —“meta-analysis” —animal —“in vitro”

**Table 2:** Demographic characteristics of included studies.

Author	Year	Location	Study Design	Sample Size	Mean Age (Years)	Male:Female Ratio	Follow-Up Period
Aaboubout et al. [14]	2021	Netherlands (single center)	Retrospective cohort, pT1–T2 cN0 OCSCC	222	(median ~64.5)	138:84	Up to 5 y (survival endpoints)
Chen et al. [15]	2024	Taiwan (national registry)	Retrospective registry, cT2N0 (AJCC-8)	4723		4205:518	NR
D’Cruz et al. [16]	2015	India (multicenter)	RCT: Elective ND vs. therapeutic ND	596 randomized (496 analyzed)	48 (20–75)	~374:122	Median 39 mo
Ebrahimi et al. [17]	2014	11 centers, 8 countries	Multicenter observational (staging cohort)	3149	(median 53)	2074:1075	Median 40 mo
Feng et al. [5]	2020	USA & China (two centers)	Retrospective cT1N0 (multiple subsites)	283	~62	158:125	≥24 mo protocol
Melchers et al. [18]	2012	Netherlands (single center)	Retrospective cohort pT1–2	212 (174 END; 38 observe)	(median ~61.5)	119:93	pN0 median 45 mo; observe median 56.5 mo
Nguyen et al. [7]	2021	New Zealand (single center)	Retrospective stage I cT1N0	70	65	40:30	Median 55 mo (4–148)
Wushou et al. [19]	2021	USA (SEER)	Retrospective registry cT1N0	5752	62.0	NR	Mean 62.4 mo
Wu et al. [20]	2022	China (single center)	Retrospective cT1N0 tongue; ultrasound-DOI guided	178	NR	NR	NR

**Table 3:** Technical characteristics relevant to DOI-guided neck management.

Author	Subsite(s) Included	AJCC-8 T-Category Basis (Clinical vs. Pathologic)	DOI Ascertainment Modality & Protocol	DOI Distribution/Strata	DOI Threshold(s) Used to Guide END (mm)	Additional Risk Factors Integrated (Grade/PNI/LVI/DOI × Subsite)	Neck Management Strategy/Arms	Reference Standard for Nodal Status	Primary Endpoint Definition
Aaboubout et al. [14]	Tongue, FOM, buccal, gingiva, lip, retromolar	Pathologic pT1–T2	Pathology per AJCC reconstructed mucosal plane	Median ~4.5 mm; bins ≤4 vs. >4	>4 (risk > 20% ≈ 4.3 mm)	Grade, diameter; PNI explored	END (ipsi/bi-level) vs. observation	Pathology in END; clinical-imaging surveillance otherwise	Occult nodes; RRFS/DSS/OS by DOI & management
Chen et al. [15]	All oral cavity subsites	Clinical cT2N0	Pathologic DOI extracted from registry reports	Continuous; optimized at 5 mm	≥5	Grade, margins, adjuvant; comorbidity	END vs. observation; adjuvant per practice	Pathologic nodal yield (median ~27)	Neck control, DSS, OS (multivariable)
D’Cruz et al. [16]	Predominantly tongue ± buccal/FOM	Clinical cT1–T2	DOI not used for allocation; standard pathology	NR	NR (trial not DOI-guided)	Covariates in adjusted models	Elective ND vs. therapeutic ND (on relapse)	Pathology in END; clinical surveillance otherwise	OS, DFS; cervical relapse
Ebrahimi et al. [17]	Oral cavity (multi-institution)	Pathologic T with DOI incorporated	Pathology; continuous DOI	Median by pT: ~5/9/13.5/15 mm (pT1–4)	5 & 10 (staging cut-points)	pT, pN, adjuvant, era; sensitivity with age/sex/ECS/margins	Not interventional (staging prognostics)	Pathologic nodal category	5-y DSS; prognostic discrimination (C-index)
Feng et al. [5]	Tongue, FOM, buccal, lower gingiva, others	Clinical cT1N0	Pathology; subsite-specific modeling	Bins: <2, 2–3, 3–4, 4–5, ≥5 mm	Subsite-specific (e.g., ≥3 FOM/BOT; ≥4 others)	Grade; LVI/PNI where available	Mixed: some END vs. observation	Pathology for END; imaging/clinical follow-up	2-y nodal metastasis risk by DOI × subsite
Melchers et al. [19]	Tongue, FOM, gum, cheek, retromolar, other	Pathologic pT1–T2	Pathology with reconstructed mucosal line	Median 6.0 mm; ROC-based	~4–4.6 (ROC 4.59; pragmatic 4)	LVI, depth; PNI considered	END vs. observation (watch-and-wait)	True nodal status (END or ≥2 y follow-up)	True N+, regional recurrence, survival
Nguyen et al. [7]	All oral cavity subsites	Clinical cT1N0 (stage I)	Pathologic DOI on resection	Per-mm; policy focus at 3 mm	≥3	Grade, PNI, LVI; DOI × features	END vs. observation; salvage as needed	Pathology (END) or relapse confirmation	Occult nodes (END) & regional relapse (observe); OS/DFS
Wushou et al. [19]	All oral cavity subsites	Clinical cT1N0	DOI not in SEER	N/A	N/A	Grade, age, sex, site, race	END vs. no END	Survival follow-up in registry; path in END	OS, DSS (population-level)
Wu et al. [20]	Tongue only	Clinical cT1N0	Intraoral ultrasound DOI; stratified	<4 vs. ≥4 mm (US-DOI)	4	Grade, PNI in models	END vs. observation	Pathology in END; clinical/imaging follow-up	5-y regional control & DSS by US-DOI × treatment

**Table 4:** GRADE summary of certainty across evidence strata.

Evidence Type (Design Group)	n (Studies)	Recurrent Signal Across the Group	Risk of Bias	Between-Study Inconsistency	Applicability (Indirectness)	Imprecision	Other Considerations	Overall Certainty (GRADE)
Randomized comparison (elective vs. therapeutic neck dissection) [16]	1	Elective treatment of the cN0 neck was associated with superior time-to-event outcomes despite not being DOI-allocated [16].	Low	Not serious (single RCT)	Some concerns (trial not DOI-guided)	Not serious (precise HRs)	Large, consistent effect	Moderate
Institutional retrospective cohorts (pathology-DOI and policy-guided) [5,7,14,18]	4	Threshold behavior clustered near 3–4 mm with improved regional control when END was used above the inflection; composite pathology refined selection [5,7,14,18].	Low to moderate	Serious (cut-points and subsites varied)	Not serious (cT1–T2N0 OCSCC)	Some concerns (single-center sizes)	Dose–response by DOI supports plausibility	Low
Ultrasound-DOI stratified cohort (oral tongue) [20]	1	Preoperative US-DOI ≥ 4 mm identified groups with better regional control under END than observation [20].	Low to moderate	Not applicable (single study)	Not serious (same target population)	Serious (single-center, modest n)	Direct preoperative triage relevance	Low
National/registry datasets (with and without explicit DOI) [15,19]	2	END favored neck control and survival; DOI ≥ 5 mm marked higher risk where available [15,19].	Low to moderate	Not serious (directionally concordant)	Serious (SEER lacked DOI granularity)	Not serious (large cohorts)	Residual confounding likely	Low to moderate
Multicenter prognostic staging cohort (DOI bands, non-interventional) [17]	1	DOI (≈5/10 mm bands) improved prognostic discrimination and underpinned AJCC-8 staging logic [17].	Low to moderate	Not serious (multi-institution)	Serious (staging rather than treatment effect)	Not serious (ample events)	Biological gradient consistent	Low

## Data Availability

The data will be available on reasonable request from the corresponding author.

## Abbreviations

The following abbreviations are used in this manuscript:

AJCC-8	American Joint Committee on Cancer, 8th edition
AUC	Area Under the Curve (ROC analysis)
BOT	Base of Tongue
CI	Confidence Interval
cN0	Clinically Node-Negative
cT1–T2	Clinical Tumor Stage 1 to 2
CT	Computed Tomography
DSS	Disease-Specific Survival
DFS	Disease-Free Survival
DOI	Depth of Invasion
ECS	Extranodal (Extracapsular) Spread
END	Elective Neck Dissection
FOM	Floor of Mouth
GRADE	Grading of Recommendations, Assessment, Development and Evaluation
HR	Hazard Ratio
LVI	Lymphovascular Invasion
MRI	Magnetic Resonance Imaging
NNT	Number Needed to Treat
NR	Not Reported
OCSCC	Oral Cavity Squamous Cell Carcinoma
OS	Overall Survival
pN	Pathologic Nodal Category
pT	Pathologic Tumor Category
PECOS	Population, Exposure, Comparator, Outcomes, Study design
PNI	Perineural Invasion
PRISMA	Preferred Reporting Items for Systematic Reviews and Meta-Analyses
RCT	Randomized Controlled Trial
RoB 2.0	Cochrane Risk of Bias 2.0 Tool
ROBINS-I	Risk Of Bias In Non-randomized Studies of Interventions
ROC	Receiver Operating Characteristic
RRFS	Regional Recurrence-Free Survival
SEER	Surveillance, Epidemiology, and End Results (U.S. registry)
SLNB	Sentinel Lymph Node Biopsy
US-DOI	Ultrasound-measured Depth of Invasion
y/mo	Years/Months
